# A meta-analysis of observational studies on anticholinergic burden and fracture risk: evaluation of conventional burden scales

**DOI:** 10.1186/s40780-021-00213-y

**Published:** 2021-09-01

**Authors:** Yukari Ogawa, Toshinori Hirai, Kiyoshi Mihara

**Affiliations:** 1 Department of Pharmacy, Faculty of Pharmacy, Musashino University, 1-1-20 Shin-machi, Nishitokyo-shi, Tokyo, 202-8585 Japan; 2 Department of Pharmacy, Faculty of Medicine, Mie University Hospital, Mie University, 2-174 Edobashi, Tsu, Mie 514-8507 Japan

**Keywords:** Anticholinergic drugs, Anticholinergic burden scale, Fracture, Meta-analysis

## Abstract

**Background:**

Anticholinergic burden potentially increases the risk of fracture. Although there are various anticholinergic burden scales, little is known about the inter-scale compatibility regarding the relationship of anticholinergic burden with fracture risk. We performed meta-analysis to examine the association of fracture risk with anticholinergic burden measured using various scales.

**Methods:**

Primary literature was retrieved from PubMed (1966 to March, 2021), the Cochrane Library (1974 to March, 2021), Scopus (1970 to March, 2021), and Ichushi-web (1983 to March, 2021). Cohort and case-control studies that evaluated the association between any fracture and anticholinergic drugs were included. Additionally, we included studies in which patients were administered anticholinergic drugs included on the anticholinergic risk scale (ARS), anticholinergic cognitive burden (ACB), anticholinergic drug scale, or drug burden index-anticholinergic component. Random effects models were used to calculate pooled relative risk (RR) and 95% confidence interval (CI) due to heterogeneity among the studies. Publication bias was examined by funnel plots and the Begg’s test.

**Results:**

A total of 49 datasets from 10 studies were included in the meta-analysis. Six of the 10 studies included only patients aged over 65 years, who accounted for 93% of the total study population (453,186/487,247). Meta-analysis indicated a positive relationship between use of anticholinergic drugs and fracture risk, regardless of the anticholinergic burden scale used. However, the relationship between anticholinergic burden and fracture risk varied depending on the scale used. Fracture risk increased linearly with increasing anticholinergic burden measured using ARS. ARS 1 point was associated with 28% increase in fracture risk, ARS 1–2 point(s) with 39%, ARS 2 points with 54%, ARS 3 points with 66%, and ARS ≥ 4 points with 77%. On the other hand, ACB 1 point and ACB 2 points were associated with similar fracture risk (pooled RR [95% CI]: overall; 1.28 [1.18–1.39], 1 point; 1.12 [1.06–1.18], 2 points; 1.15 [1.08–1.23]).

**Conclusions:**

This result suggests that the relationship between anticholinergic drug burden and fracture risk may differ depending on the anticholinergic burden scale used.

**Supplementary Information:**

The online version contains supplementary material available at 10.1186/s40780-021-00213-y.

## Background

Anticholinergic drugs act on the muscarinic receptors in central and peripheral nervous systems and inhibit acetylcholine-mediated responses by binding to these receptors. A study of the trend of anticholinergic drug prescriptions from 1995 to 2010 reported an increase in prescriptions of these agents from 20.7% in 1995 to 23.7% in 2010 [1] . Anticholinergic drugs comprise drugs with a broad spectrum of physiological effects, including psychotropic drugs, antiparkinsonian drugs, drugs for overactive bladder, and some antiarrhythmic drugs (such as disopyramide). Therefore, patients who receive prescriptions from more than one physician potentially have an increased anticholinergic burden due to concomitant use of anticholinergic drugs. The Beers criteria [2], the STOPP/START criteria [3], and the Japanese Guidelines for Medical Treatment and its Safety in the Elderly [4] recommend reassessment for appropriate use of anticholinergic drugs that can induce dry mouth, constipation, blurred vision, and cognitive dysfunction in older patients. According to some previous studies [5–7], olanzapine and paroxetine users have 1.49-fold and 1.21-fold, respectively, higher risk of fracture compared with non-users. In contrast, other studies found no significant fracture risk of anticholinergic drugs [8, 9]. Therefore, whether the use of anticholinergic drugs increases the fracture risk remains controversial.

A survey of 488,759 cases of hip fractures in Japan found that approximately 80% of fracture events were caused by accidental falls, and the number of fractures increased over time and tended to increase with age [10]. According to the Ministry of Health, Labour and Welfare comprehensive survey of living conditions in 2019, the number of people who needed nursing care due to a fracture or fall was the third highest among people requiring care, and accounted for 12.0% of the total number of people requiring care [11]. Fractures and falls reduce quality of life due to pain and loss of motor function [12]. As of 2020, 28.7% of the total population in Japan were aged 65 and above, and 14.9% were aged 75 and over [13]. It is important to implement risk management to avoid fractures and falls in Japan with a super-aged population.

Various scales have been developed to assess the anticholinergic burden, such as the anticholinergic risk scale (ARS) [14], the anticholinergic cognitive burden (ACB) [15, 16], the anticholinergic drug scale (ADS) [17], and the drug burden index-anticholinergic component (DBI-Ach) [18]. Many studies have reported the relationship between anticholinergic burden measured using various scales and fall-related fractures [19–21], though the direct causal mechanism of anticholinergic effect on fractures has not been proved to date. Reinold et al. [21] reported an association between anticholinergic burden and increased risk of fractures with possible dose-exposure gradient in studies using ARS. However, it remains unclear whether the same trend between anticholinergic burden and fracture risk is observed when using other anticholinergic burden scales such as ACB, ADS and DBI-Ach. However, several reports have pointed out the discrepancy of risk scores assigned to drugs when using various anticholinergic burden scales [22–24]. For instance, these studies showed that the kappa values between ARS and ACB ranged from 0.25 to 0.43 (i.e., low consistency) [22–24]. It is important to address the discrepancies among anticholinergic burden scales which would affect the assessment of fracture risk. To our knowledge, no studies have systematically analyzed whether using different scales for calculating anticholinergic burden affects fracture risk assessment. In this study, we performed meta-analysis aiming to elucidate the association between fracture risk and anticholinergic burden measured using four widely used anticholinergic burden scale; namely, ARS, ACB, ADS and DBI-Ach.

## Methods

### Data sources and searches

We conducted meta-analysis in accordance with the Preferred Reporting Items for Systematic Reviews and Meta-analyses statement [25]. To assemble all of the relevant published studies and unpublished literature, the public databases used in the literature search were PubMed (1966 to March, 2021), the Cochrane Library (1974 to March, 2021), Scopus (1970 to March, 2021), and Ichushi-web (1983 to March, 2021). We combined the MeSH terms or keywords including “anticholinergic^*^”, “drug burden index”, “cholinergic antagonists”, “fracture^*^” and “fractures, bone”. In addition, we manually searched the reference lists in all the selected studies and related articles.

### Study selection

The inclusion criteria of the present study were: (1) cohort studies or case-control studies that evaluated the association between anticholinergic drugs and fracture risk; (2) studies in which patients were administered anticholinergic drugs defined by ARS, ACB, ADS or DBI-Ach; (3) fracture was defined by objective measures such as the International Classification of Diseases; (4) the association between anticholinergic drugs and fracture was assessed using either the hazard ratio (HR), risk ratio (RR) or odds ratio (OR). We included studies in which 95% confidence intervals (CI) were not listed, provided that the graphs were visually decipherable. Studies including patients younger than 15 years of age were excluded. Since we focused on the real-world data to evaluate the association of anticholinergic burden with fracture event, interventional studies, such as randomized controlled trials, were excluded. Duplicated studies, including different report using same population, were excluded. Two investigators (YO and TH) screened the articles independently using the inclusion and exclusion criteria. When there was a disagreement between the two investigators, a final decision was made after careful discussion.

### Data extraction

Study design (cohort study, case-control study), number of patients, sex of patients, age of patients, names of anticholinergic drugs or scores on anticholinergic burden scales, country in which the study was performed, anatomical site of fracture, follow-up period, and confounders were extracted from each study. In addition, for studies that did not document the anticholinergic burden scores in the manuscript but identified the names of anticholinergic agents, we manually calculated anticholinergic burden scores using ARS, ACB and ADS. The confounder-adjusted RR was used as a measure of the association between use of anticholinergic drugs and fracture risk. In case a study had reported stratified population data, we used all the datasets for the meta-analysis unless patient overlap existed.

### Assessment of risk of bias in included studies

The quality of each study was assessed using the risk of bias assessment tool for non-randomized studies (RoBANS) [26]. The tool consists of six categories: selection of patients, confounding variables, measurement of exposure, blinding of outcome assessments, incomplete outcome data, and selective outcome reporting. According to RoBANS, we assessed the risk of bias as low, high, or unclear based on adjustment of age and comorbidities, objective measurement of fracture event, and missing data. Then overall risk of bias was assigned to each study as follows; included studies that were categorized as having an overall low risk of bias (≤ 1 category evaluated as having high or unclear risk of bias), medium risk of bias (two categories evaluated as having high or unclear risk of bias) or high risk of bias (≥ 3 categories evaluated as having high or unclear risk of bias) [27].

### Comparison between ARS and ACB for fracture risk in the same cohort

ARS and ACB were frequently used for calculation of anticholinergic burden in the included studies, whereas ADS and DBI-Ach were less commonly used. Thus, we compared whether there was a difference in the estimated fracture risk related to anticholinergic burden calculated using ARS and ACB in the same cohort. Among the included studies, if both ARS and ACB scores could be calculated within the same cohort of individuals, we used them for performing meta-analysis to evaluate the relationship of fracture risk with anticholinergic burden calculated using ARS and ACB separately. Based on the RR for fracture risk calculated in our meta-analysis, we classified them into five categories: low fracture risk (RR 1.0 to 1.2), medium/low risk (RR 1.2 to 1.4), medium risk (RR 1.4 to 1.6), medium/high risk (RR 1.6 to 1.8), and high risk (RR 1.8 and higher). Then, we examined the concordance between ARS and ACB with respect to the relationship between anticholinergic burden score and risk category.

### Data synthesis and analysis

The association between anticholinergic drugs and fracture events was assessed by RR and 95% CI. Because the absolute value of fracture risk is small, OR was judged to be comparable to RR [28, 29]. Therefore, for case-control studies, OR was replaced by RR as an alternative value for data analysis. We judged HR was comparative to RR under proportional hazard assumption [30]. When there were two or more included studies for an endpoint, we performed a meta-analysis on the studies and calculated the integrated RR and 95% CI using random effects methods (Mantel-Haenszel). Inter-study heterogeneity was assessed by I^2^ statistic (I^2^ ≥ 75% indicates substantial heterogeneity) [31]. Publication bias was examined by funnel plots and the Begg’s test [32]. Funnel plots were constructed by plotting RR as effect size estimate on the horizontal axis and the standard error of log RR as sample size on the vertical axis, and whether the distribution was symmetrical was determined visually. Furthermore, publication bias was judged to be present when the Begg’s test yielded *P* <  0.05. A subgroup analysis was conducted in elder (65 years and over) patients. Data were analyzed using Stata15 (College Station, TX, USA). A *P* value less than 0.05 was considered significant.

## Results

### Study retrieval and characteristics of included studies

**Fig. 1:**
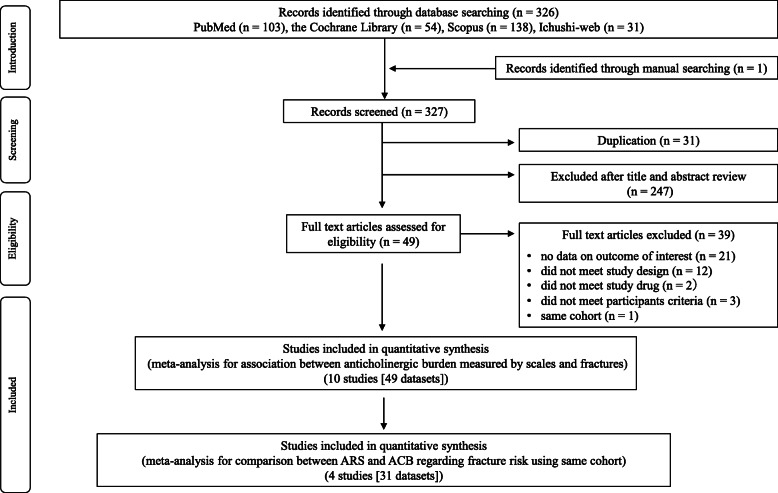
Study selection flow diagram. n: total number of studies

The primary literature search retrieved 327 studies comprising 103 from PubMed, 54 from Cochrane Library, 138 from Scopus, 31 from Ichushi-web, and 1 from manual search (Fig. 1). Two investigators (YO and TH) independently reviewed the articles using the inclusion and exclusion criteria and finally included 10 studies [19, 20, 33–40]. A summary of the studies analyzed is shown in Table 1 and Table 2. The total sample size was 487,247 patients. Patients in five [20, 33, 35, 37, 39] of the ten studies were aged 65 years and older. Three [20, 36, 37] of the ten studies were conducted in Asia, but none of them were conducted in Japan. Regarding study design, eight studies were cohort studies [20, 33–37, 39, 40] and two were case-control studies [19, 38]. Seven [20, 33, 34, 37–40] of the ten studies utilized ARS to calculate anticholinergic exposure. Six [20, 33, 35, 36, 39, 40] of the ten studies utilized ACB to calculate anticholinergic burden. In all the included studies, the confounders used in the analyses of fracture risk ratios were mainly age, sex, comorbidities, and concomitant medications. We retrieved a total of 49 datasets from the included studies for performing meta-analysis. Five studies [19, 20, 34, 37, 38] had shown OR as the fracture risk indicator, and the incidence of fractures among the anticholinergic group in these studies was around 10% except for the study of Lu et al. (25.7%) [37]. Results of assessment for risk of bias of individual studies are shown in Table 3. Analysis using RoBANS indicated an overall low risk of bias in all the studies except the study of Ishida et al. [35].

**Table 1:** Characteristics of cohort studies included in the present meta-analysis

Study	Country	Sample size (AC drug user, (n))	Female (%)	Age	Anatomical site of fracture	AC burden scale	Mean follow-up	Adjusted for confounders
Bali et al. (2016) [33]	USA	9240 (4620)	67	≥ 65 yr	hip	ARS, ACB, ADS*	2.0 years	age, sex, race, co-medications, and illness history during 1-year baseline period
Crispo et al. (2016) [34]	Canada	16,302 (13,839)	47	≥ 70 yr: 82.3%	any fracture	ARS	3–6 days: 2463 persons 7–30 days: 5799 persons ≥31 days: 141 persons	age, sex, race, length of stay, Elixhauser comorbidity score, census region, urban/rural status, hospital size (number of beds), and hospital teaching status
Hsu et al. (2017) [20]	Taiwan	116,043 (43,301)	50	≥ 65 yr	any fracture	ARS, ACB, DBI-Ach	8.3 years	sex and time-varying comorbidities (annually measured by Carlson Comorbidity Index)
Ishida et al. (2019) [35]	USA	60,007 (3745)	56	≥ 65 yr	hip, femur, pelvis, foot, arm, hand, or axial skeleton	ACB**	243 days******	age, sex, race, duration on dialysis, network, BMI, alcohol dependence, coronary artery disease, cancer, other cardiac disease, dysrhythmia, congestive heart failure, cerebrovascular disease, diabetes, drug dependence, opioid dependence, hypertension, inability to ambulate, inability to transfer, chronic obstructive pulmonary disease, peripheral vascular disease, tobacco dependence, dementia, depression, seizure/epilepsy liver disease, medication burden, and concomitant medications
Kao et al. (2018) [36]	Taiwan	14,635 (2927)	69	52.0 ± 16.9 yr (study cohort) 51.9 ± 17.1 yr (control)	any fracture	ACB***	3.0 years	monthly income, geographical region, urbanization level, and comorbidities
Lu et al. (2015) [37]	Taiwan	59,042 (7461)	49	≥ 65 yr	any fracture	ARS	7.95 ± 3.03 years	age, sex, and time-varying comorbidities
Moga et al. (2013) [39]	USA	6594 (1125)	4	≥ 65 yr	hip or any fracture	ARS, ACB, ADS****	AC drug users: 49 days (median) AC drug nonusers: 95 days (median)	demographic characteristics, continence status (bladder and bowel), continence management, preexistent urinary tract infections, body mass index, comorbidities, other medication use, cognitive status, mobility at baseline
Sørensen et al. (2013) [40]	Denmark	2224 (1216)	not mention	68.6 ± 12.8 yr	hip	ARS, ACB, ADS*****	not mention	age at diagnosis (schizophrenia), sex, alcohol misuse, somatic score

AC: anticholinergic, ARS: anticholinergic risk scale, ACB: anticholinergic cognitive burden, ADS: anticholinergic drug scale

*AC drug used was paroxetine, which is 1 point on ARS, 3 points on ACB, and 1 point on ADS

** AC drugs used were amitriptyline, paroxetine, doxepin, nortriptyline, imipramine, desipramine and clomipramine, which are 3 points on ACB; and protriptyline which is not listed on ACB. Since less than 10 (0.01%) patients were taking protriptyline, we categorized all drugs use as ACB 3 points

*** AC drugs used were oxybutynin, trospium, tolterodine, solifenacin and propiverine, which are 3 points on ACB

**** AC drug used was oxybutynin, which is 3 points on ARS, ACB, and ADS

***** AC drugs used included risperidone, which is1 point on ARS and ACB; quetiapine which is 1 point on ARS and 3 points on ACB; olanzapine, which is 2 points on ARS, 3 points on ACB and 1 point on ADS; and aripiprazole, which is 1 point on ACB

******Follow-up period was calculated using fracture rate (6 events per 100 person-years) and number of fractures (4% of the cohort) described in the article

**Table 2:** Characteristics of case-control studies included in the present meta-analysis

Study	Country	Sample size (case/control)	Age	Female (%)	Anatomical site of fracture	AC burden scale	AC exposure	Adjusted for confounders
Chatterjee et al. (2016) [19]	USA	202,260 (40,452/161,808)	81.1 ± 7.4 yr	85	hip or femur	ADS	prescribed 30–90 days before fracture	age, sex, medications, race, medications (cardiovascular drugs, antidepressants, anticonvulsants, antipsychotics, and benzodiazepines), and comorbidities (dementia, mood disorders, anxiety, schizophrenia, Parkinson’s disease, insomnia, cerebrovascular events, osteoarthritis, osteoporosis, rheumatoid arthritis), and duration of depression
Machado-Duque et al. (2018) [38]	Colombia	900 (300/600)	81.6 ± 8.9 yr	71	hip	ARS	prescribed 30 days before fracture	use of statins, proton pump inhibitors, corticosteroids, oral antidiabetics, polypharmacy (≥ 5 drugs), and treatment city

*AC* anticholinergic, *ARS* anticholinergic risk scale, *ADS* anticholinergic drug scale

**Table 3:** Quality assessment using risk of bias assessment tool for non-randomized studies

Study	Selection of participants	Confounding variables	Measurement of exposure	Blinding of outcome assessments	Incomplete outcome data	Selective outcome reporting	Overall risk of bias
Bali et al. (2016) [33]	unclear	low	low	low	low	low	low
Chatterjee et al. (2016) [19]	low	low	low	low	low	unclear	low
Crispo et al. (2016) [34]	unclear	low	low	low	low	low	low
Hsu et al. (2017) [20]	unclear	low	low	low	low	low	low
Ishida et al. (2019) [35]	unclear	low	low	low	unclear	low	medium
Kao et al. (2018) [36]	low	low	low	low	unclear	low	low
Lu et al. (2015) [37]	unclear	low	low	low	low	low	low
Machado-Duque et al. (2018) [38]	low	low	low	low	low	high	low
Moga et al. (2013) [39]	low	low	low	low	low	low	low
Sørensen et al. (2013) [40]	low	low	low	low	low	low	low

### Association between anticholinergic burden and fracture risk

#### ARS

**Fig. 2:**
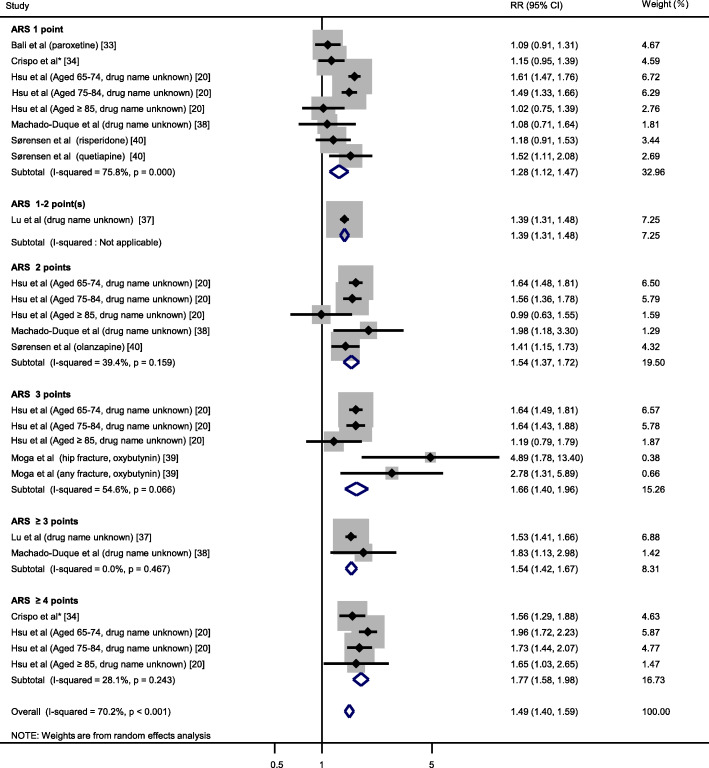
Forest plot of meta-analysis of fracture risk associated with anticholinergic burden using ARS. Gray box (■) represents sample size in each study. Risk ratio (RR) and 95% confidence interval (CI) are shown. The analysis was performed using Mantel-Haenszel method with random effects model. *Drugs with ARS 1 point used were carbidopa-levodopa (60.7%), quetiapine (9.6%), metoclopramide (8.7%), pramipexole (8.4%), haloperidol (6.4%), entacapone (6.3%), risperidone (5.1%), mirtazapine (4.9%), paroxetine (4.4%), trazodone (4.1%), ranitidine (3.5%), selegiline (2.5%), ziprasidone (0.9%), and methocarbamol (0.6%). Drugs with ARS 2 points used were olanzapine (4.6%), amantadine (4.1%), tolterodine (3.3%), loratadine (2.1%), prochlorperazine (2.1%), loperamide (1.8%), cyclobenzaprine (1.3%), nortriptyline (0.6%), cetirizine (0.5%), clozapine (0.4%), cimetidine (0.3%), desipramine (0.1%), and pseudoephedrine (< 0.01%). Drugs with ARS 3 points used were diphenhydramine (7.6%), promethazine (6.1%), oxybutynin (3.4%), atropine (2.6%), hydroxyzine (2.4%), benztropine (2.2%), amitriptyline (1.7%), meclizine (1.5%), hyoscyamine (0.6%), dicyclomine (0.6%), tizanidine (0.3%), chlorpromazine (0.3%), perphenazine (0.3%), cyproheptadine (0.3%), imipramine (0.2%), carisoprodol (0.2%), thioridazine (0.1%), chlorpheniramine (0.1%), fluphenazine (0.1%), trifluoperazine (0.1%), and thiothixene (0.1%)

A total of 25 datasets from 7 studies [20, 33, 34, 37–40] were included in the meta-analysis for ARS. Forest plot of all RRs of fracture risk associated with anticholinergic drugs in individual studies and overall RR are shown in Fig. 2. The fracture risk was significantly higher in the anticholinergic group compared to the non-anticholinergic group (pooled RR [95% CI]: 1.49 [1.40–1.59]). Furthermore, the risk of fractures increased linearly as anticholinergic burden increased. ARS 1 point was associated with 28% increase in fracture risk, ARS 1–2 point(s) with 39%, ARS 2 points with 54%, ARS 3 points with 66%, and ARS ≥ 4 points with 77%.

#### ACB

**Fig. 3:**
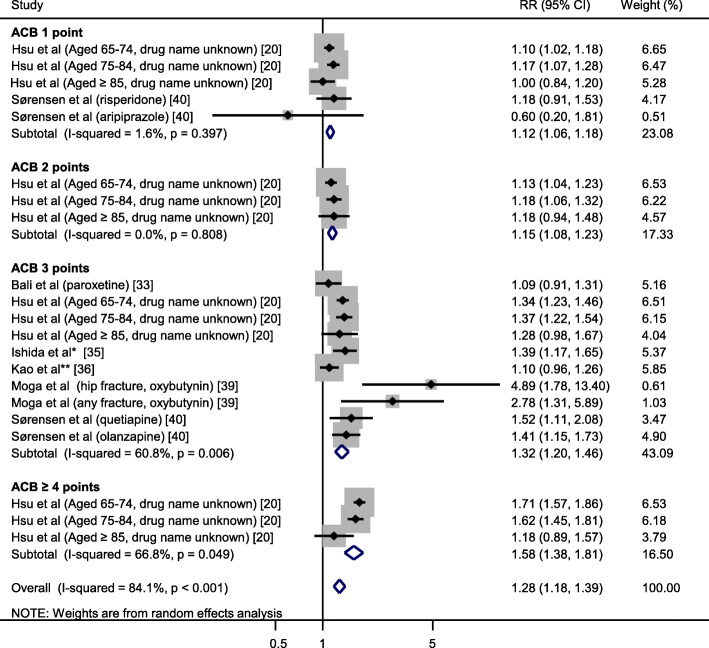
Forest plot of meta-analysis of fracture risk associated with anticholinergic burden using ACB. Gray box (■) represents sample size in each study. Risk ratio (RR) and 95% confidence interval (CI) are shown. The analysis was performed using Mantel-Haenszel methods with random effects model. *Anticholinergic drugs used were amitriptyline (3%), paroxetine (2%), doxepin (0.6%), nortriptyline (0.5%), imipramine (0.1%), desipramine (0.04%), clomipramine (0.01%), and protriptyline (0.01%). All drugs except protriptyline are listed as ACB 3 points. However, since few patients used protriptyline, we categorized all anticholinergic drugs used as ACB 3 points. **Anticholinergic drugs used were oxybutynin, trospium, tolterodine, solifenacin, and propiverine. All drugs are listed as ACB 3 points

A total of 21 datasets from 6 studies [20, 33, 35, 36, 39, 40] were included in the meta-analysis for ACB. Forest plot of all RRs of fracture risk associated with anticholinergic drugs in individual studies and overall RR are shown in Fig. 3. The fracture risk was significantly higher in the anticholinergic group compared to the non-anticholinergic group (pooled RR [95% CI]: 1.28 [1.18–1.39]). ACB 1 point and ACB 2 points were associated with similar fracture risk (pooled RR [95% CI]: 1.12 [1.06–1.18] and 1.15 [1.08–1.23], respectively). On the other hand, ACB 3 points was associated with 32% increase in fracture risk, and ACB ≥ 4 points with 58%.

#### ADS

A total of 6 datasets from 4 studies [19, 33, 39, 40] were included in the meta-analysis of ADS. Forest plot of all RRs of fracture risk associated with anticholinergic drugs in individual studies and overall RR are shown in Additional file 1. The fracture risk was significantly higher in the anticholinergic group compared to the non-anticholinergic group (pooled RR [95% CI]: 1.19 [1.08–1.31]). The fracture risk was comparable for ADS 1 point and ADS 2 points, although only one dataset [40] for available for ADS 2 points.

#### DBI-Ach

The study of Hsu et al. [20] assessed the fracture risk and anticholinergic burden using DBI-Ach, and DBI-Ach scores were divided into two categories (0 < DBI-Ach ≤ 0.5 and 0.5 < DBI-Ach ≤ 1). We performed meta-analysis using 6 datasets. The fracture risk was significantly higher in the anticholinergic group compared to the non-anticholinergic group (pooled RR [95% CI]: 1.46 [1.36–1.57]). Fracture risk showed a linear increase with increase in anticholinergic burden (pooled RR [95% CI] for 0 < DBI-Ach ≤ 0.5: 1.39 [1.28–1.51], for 0.5 < DBI-Ach ≤ 1: 1.60 [1.47–1.73]). This study included only patients aged 65 years and older.

#### Subgroup analysis

We next focused on elder patients aged 65 years and older. In the subgroup analysis, we included six studies that investigated only patients aged 65 years and older [20, 33, 35, 37, 39] together with the study of Chatterjee et al. [19] that included patients with mean age of 81.1 ± 7.4 years, and we estimated that 95% of the eligible patients were older than 65 years. Additional file 2 summarizes the pooled RRs associated with anticholinergic drugs in elder patients. For ARS, ACB and ADS, fracture risk was higher in users of anticholinergic drugs compared to non-users of anticholinergic drugs (pooled RR [95% CI]: 1.53 [1.42–1.65], 1.29 [1.17–1.41] and 1.15 [1.04–1.27], respectively). The results for DBI-Ach in elder patients are described above.

#### Publication bias

Funnel plots of the study data for the four anticholinergic burden scales used in meta-analysis are shown in Additional file 3. Regarding ADS, the funnel plot was asymmetric and the result of the Begg’s test showed statistically significant publication bias (*P* = 0.039).

### Comparison between ARS and ACB for fracture risk in same cohort

**Fig. 4:**
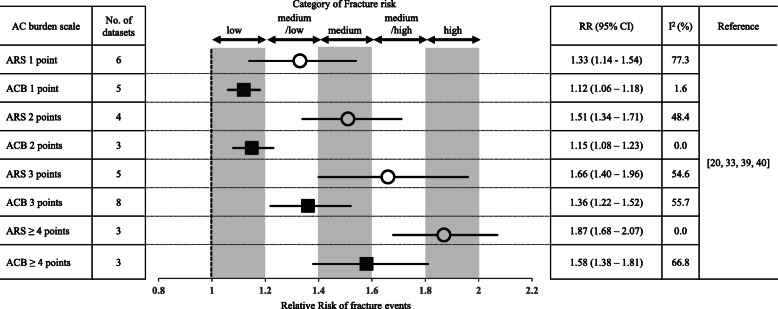
Summary of results of meta-analyses including four studies that measured anticholinergic burden using both ARS and ACB

Four studies [20, 33, 39, 40] utilized both ARS and ACB to measure anticholinergic burden within the same cohort. We used the cohort data in which both anticholinergic burden scales were used simultaneously to validate the result of our meta-analysis. Figure 4 presents a summary of the individual meta-analyses performed. A linear relationship was found between fracture risk and anticholinergic burden measured by ARS, but not ACB. In addition, RRs of ARS 1 point and ACB 3 points were comparable (pooled RR [95% CI]: 1.33 [1.14–1.54] and 1.36 [1.22–1.52], respectively). The drugs used in ARS 1-point group were paroxetine, risperidone and quetiapine; while the drugs used in ACB 3-point group were paroxetine, oxybutynin, quetiapine and olanzapine (the study of Hsu et al. [20] did not mention the drugs in detail). Forty-nine drugs are included in ARS [14] and 99 drugs in ACB [15, 16], 35 of which are included in both ARS and ACB. Additional file 4 shows a summary of the 35 overlapping drugs. Of the 35 drugs, 22 (62.9%) showed concordance between ARS and ACB scores, but all the 22 drugs showed different fracture risk categories when using ARS and ACB. Metcavamol, paroxetine, and quetiapine were in the same fracture risk category, but these drugs scored differently in ARS and ACB (ARS: 1 point, ACB: 3 points). Of the 22 drugs with concordant ARS–ACB scores, 18 had a 2-rank difference in fracture risk when using the two scales (for example, amantadine: “medium” fracture risk using ARS, “low” fracture risk using ACB). In addition, the 14 drugs listed on ARS, not on ACB, included 4 (28.6%) with 3 points [carisoprodol (not approved in Japan), fluphenazine, thiothixene (not approved in Japan) and tizanidine], while the 64 drugs listed on ACB, not on ARS, included 45 (70.3%) with 1 or 2 points (ACB 1 point: 36 drugs, ACB 2 points: 9 drugs) and were categorized as “low” fracture risk.

## Discussion

This study found that regardless of which anticholinergic burden scale was used to assess anticholinergic burden, anticholinergic drug users had significantly increased fracture risk by 19 to 49% compared to non-users. Interestingly, however, the relationship between anticholinergic burden and fracture risk showed different trends depending on the anticholinergic burden scale used. Reinold et al. [21] have reported an association of anticholinergic burden with increased risk of fractures with possible dose-exposure gradient in studies using ARS. Our meta-analyses suggested that the risk of fractures increased dose-dependently in studies using ARS as well as in a single study using DBI-Ach. Our analysis on DBI-Ach included 3 datasets with different age groups from the same study. Therefore, its extrapolation to broader population could be limited. ACB exhibits a trend different from other anticholinergic burden scales. When using ACB, although high anticholinergic burden (3 or 4 points) was associated with higher fracture risk than low anticholinergic burden (1 or 2 points), fracture rate did not differ between ACB 1 point and 2 points (RR: 1.1 and 1.15, respectively).

ARS, ACB, DBI-Ach, and ADS are widely used to assess the anticholinergic burden. In ARS, drugs are rated from 0 (no or low risk of anticholinergic adverse effects) to 3 (high potential risk). In ACB, drugs with possible anticholinergic effects are rated from 1 (no known clinically relevant negative cognitive effects) to 3 (clinically relevant negative cognitive effects). ADS ranks the anticholinergic effects of drugs in a 4-point scale of 0 (no known anticholinergic activity) to 3 (significantly marked anticholinergic activity). For each of these anticholinergic burden scales (ARS, ACB and ADS), the total anticholinergic drug exposure for an individual is the sum of the scores for all drugs. The DBI is a pharmacological risk assessment tool that calculates exposure to both anticholinergic (DBI-Ach) and sedative drugs. DBI is based on the principle of cumulative exposure and dose response.

In the included studies, ARS and ACB were commonly used to calculate anticholinergic burden. Therefore, using the same cohort, we examined whether fracture risk differs when ARS and ACB are used to measure anticholinergic burden. We divided fracture risk into five categories based on the results of our meta-analyses, ranging from “low” to “high” (Fig. 4). A previous study has reported that less than 50% of patients were classified into the same burden category by different scales including ARS and ACB, and that the level of anticholinergic burden varies depending on the assessment scale used [24]. In our analysis, ACB 1 point and ACB 2 points were both associated with “low” fracture risk. On the other hand, ARS 1 point and ACB 3 points were both associated with “medium/low” fracture risk. The discrepancy between ARS and ACB may influence the risk assessment for preventing fracture events. Of 35 drugs included in both ARS and ACB, 22 drugs have concordant ARS–ACB scores, while none of these drugs are concordant in category of fracture risk (Additional file 4). On the other hand, three drugs (methocabamol, paroxetine and quetiapine) that are in the same category of fracture risk have different scores in ARS and ACB. ARS was developed to predict the risk of anticholinergic adverse effects such as falls, dry mouth, dry eyes, dizziness, and confusion [14]. On the other hand, ACB was developed to predict the risk of cognitive impairment [15, 16]. The different methodology by which these scales were developed may give rise to the discrepancy in fracture risk prediction.

In general, pharmacokinetic changes such as decreased hepatic and renal clearance and pharmacodynamic changes such as increased sensitivity to anticholinergic drugs occur in elder people. Therefore, this population is expected to be at increased risk of drug interactions and adverse effects. In the subgroup of patients aged 65 years or older, our study showed that those who used anticholinergic drugs had increased fracture risk compared to non-users of anticholinergic drugs, regardless of the anticholinergic burden scale used. In this study, the result of meta-analysis for the elder population did not show remarkably higher fracture risk when compared to the overall result of the total study population. This finding may reflect that the overall result of our meta-analysis was derived mainly from elder patients, because 6 [19, 20, 33, 35, 37, 39] of 10 studies included only patients aged over 65 years, who accounted for 93% of the total study population (453,186/487,247). The study of Kao et al. [36] included younger patients (mean age 52 ages) than the subjects in the other studies. However, the reported RR in the study of Kao et al. [36] was comparable with those in other studies (Fig. 3).

The meta-analyses in this study showed that heterogeneity tended to be high (e.g., I^2^ 70.2% in Fig. 2, I^2^ 84.1% in Fig. 3). As visual inspection from the forest plot, the study reported by Moga et al. [39] seemed to show higher fracture risk compared with the other studies. The major differences are the shorter observation period (49 days for anticholinergic users and 95 days for non-users) and the smaller proportion of women (4%) in the study of Moga et al. [39] compared to the others. However, we could not find any accountable relationships between higher fracture risk and either shorter observation or smaller proportion of women.

Five [19, 33, 38–40] of the ten included studies focused on the risk of hip or femur fractures, the biggest concern during fall accidents, in anticholinergic drug users compared to non-users. Psychotropics, a typical class of anticholinergic drugs, are well known to cause falls [41]. Our result indicates that anticholinergic drugs may increase not only the risk of falls, but also the risk of fractures. A study of Japanese older population has reported that anticholinergic burden according to ARS was associated positively with the risk of hip or femur fractures [42]. Our finding suggests the same trend, although no Japanese studies were included in our meta-analysis.

Our study had several limitations. First, we were unable to evaluate whether the nursing and care settings were comparable in all the studies included in the meta-analysis. In the case of fall-related fractures, it is important to ensure that the living environment does not induce falls and to establish a preventive system for early detection of falls. We cannot completely exclude the possibility that environmental setting has a confounding effect on the association between anticholinergic drugs and fracture risk. Second, we did not evaluate the association between decreased bone density or a history of fractures and fracture risk. Decreased bone density and a history of fractures are risk factors for fractures [43]. Thus, we need to consider the possibility of confounding when interpreting the results of this study. None of the studies mentioned bone density of the study population, although six studies described the number of people with osteoporosis and the number of people taking osteoporosis drugs [19, 20, 33, 36–38]. The proportion of these patients varied from 1.7 to 34.3% in the six studies, although four of the six studies adjusted for a history of osteoporosis as a confounder in their analyses [19, 33, 36, 37]. Additionally, three cohort studies clearly stated that people with a history of bone fractures were excluded from the study [19, 36, 40]. Third, in the lists of ARS and ACB, 28 drugs are not approved in Japan because both scales were developed in the USA. On the other hand, drugs that are approved in Japan but not in the USA, such as eperisone, were not evaluated in the development of these scales. Forth, we were unable to retrieve the information about the anticholinergic doses and concomitant drugs from the included studies. Not only anticholinergic drugs, but also sedatives such as benzodiazepines have been reported to be risk factors for inducing fractures [44]. Finally, the number of studies included in the meta-analysis was small, thus limiting the validity of the result.

## Conclusions

Anticholinergic drug use increases fracture risk overall. However, the relationship between the anticholinergic burden and fracture risk may differ depending on the anticholinergic burden scale used. We propose that healthcare professionals should comprehensively assess the prescribed anticholinergic drugs with physicians to prevent the risk of fractures.

## Supplementary Information

**Additional file 1.** Forest plot of meta-analysis of fracture risk associated with anticholinergic burden using ADS.

**Additional file 2.** Summary of results for patients aged 65 years and above.

**Additional file 3.** Funnel plots of the meta-analyses of fracture risk associated with anticholinergic burden: a) ARS, b) ACB, c) ADS, d) DBI-Ach.

**Additional file 4.** Thirty-five drugs included in both ARS and ACB.

## Data Availability

The datasets used and/or analyzed during the current study are available from the corresponding author on reasonable request.

## Abbreviations

ACB: anticholinergic cognitive burden
ADS: anticholinergic drug scale
ARS: anticholinergic risk scale
CI: confidence interval
DBI-Ach: drug burden index-anticholinergic component
HR: hazard ratio
OR: odds ratio
RoBANS: risk bias assessment tool for non-randomized studies
RR: risk ratio
