# Supplementary material for: A meta-analysis of observational studies on anticholinergic burden and fracture risk: evaluation of conventional burden scales
Source: J Pharm Health Care Sci. 2021 Sep 1;7:30. doi: 10.1186/s40780-021-00213-y (PMC8408921; doi:10.1186/s40780-021-00213-y)
Supplement: Supplementary file 1 — Additional file 1. Forest plot of meta-analysis of fracture risk associated with anticholinergic burden using ADS. [file 40780_2021_213_MOESM1_ESM.docx]

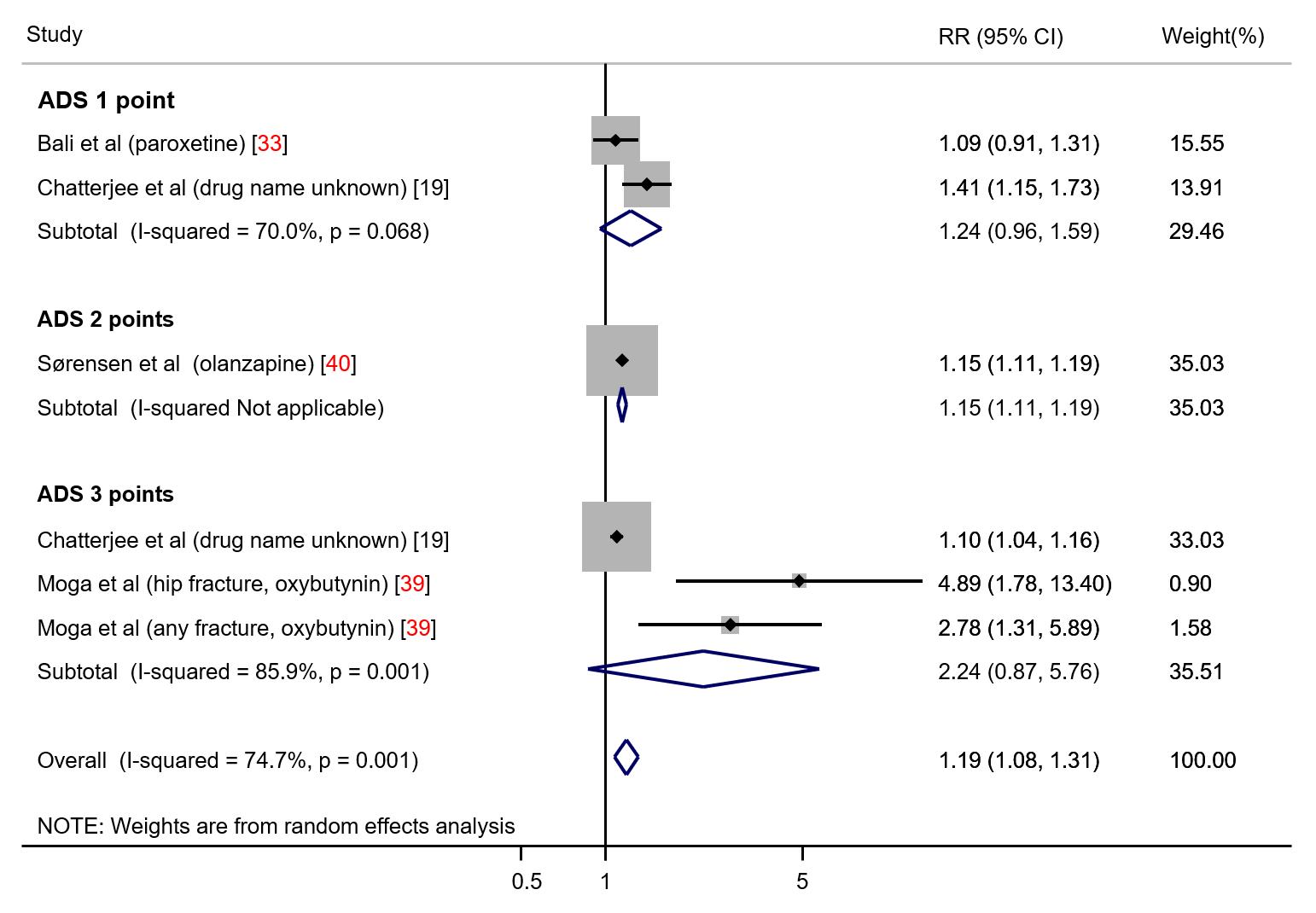


**Additional file 1** Forest plot of meta-analysis of fracture risk associated with anticholinergic burden using ADS.

ADS: anticholinergic drug scales, RR: risk ratio.

Gray box (■) represents sample size in each study. Risk ratio (RR) and 95% confidence interval (CI) are shown. The analysis was performed using Mantel-Haenszel method with random effects model.
