# Supplementary material for: A meta-analysis of observational studies on anticholinergic burden and fracture risk: evaluation of conventional burden scales
Source: J Pharm Health Care Sci. 2021 Sep 1;7:30. doi: 10.1186/s40780-021-00213-y (PMC8408921; doi:10.1186/s40780-021-00213-y)
Supplement: Supplementary file 2 — Additional file 2. Summary of results for patients aged 65 years and above. [file 40780_2021_213_MOESM2_ESM.docx]

| AC burden scale | Number of datasets | Pooled RR (95% CI) | *I^2^* statistics (%) | Reference |
| --- | --- | --- | --- | --- |
| ARS |  |  |  |  |
| overall | 17 | 1.53 (1.42-1.65) | 74.9 | [20, 33, 34, 37, 38, 39, 40] |
| ARS 1 point | 4 | 1.32 (1.10-1.60) | 84.5 |  |
| ARS 1-2 point(s) | 1 | 1.39 (1.31-1.48) | - |  |
| ARS 2 points | 3 | 1.53 (1.32-1.78) | 57.4 |  |
| ARS 3 points | 5 | 1.66 (1.40-1.96) | 54.6 |  |
| ARS ≥ 3 points | 1 | 1.53 (1.41-1.66) | - |  |
| ARS ≥ 4 points | 3 | 1.87 (1.68-2.07) | 0.0 |  |
| ACB |  |  |  |  |
| overall | 16 | 1.29 (1.17-1.41) | 87.2 | [20, 33, 35, 36, 39, 40] |
| ACB 1 point | 3 | 1.11 (1.04-1.19) | 25.2 |  |
| ACB 2 points | 3 | 1.15 (1.08-1.23) | 0.0 |  |
| ACB 3 points | 7 | 1.35 (1.20-1.51) | 60.3 |  |
| ACB ≥ 4 points | 3 | 1.58 (1.38-1.81) | 66.8 |  |
| ADS |  |  |  |  |
| overall | 5 | 1.15 (1.04-1.27) | 74.3 | [19, 33, 39, 40] |
| ADS 1 point | 1 | 1.09 (0.91-1.31) | - |  |
| ADS 2 points | 1 | 1.15 (1.11-1.19) | - |  |
| ADS 3 points | 3 | 2.24 (0.87-5.76) | 85.9 |  |

**Additional file 2** Summary of results for patients aged 65 years and above.

AC: anticholinergic, ARS: anticholinergic risk scale, ACB: anticholinergic cognitive burden, ADS: anticholinergic drug scale

Chatterjee et al. [19] was included because we estimated that 95% of the study population were aged over 65 years.
