# Supplementary material for: A meta-analysis of observational studies on anticholinergic burden and fracture risk: evaluation of conventional burden scales
Source: J Pharm Health Care Sci. 2021 Sep 1;7:30. doi: 10.1186/s40780-021-00213-y (PMC8408921; doi:10.1186/s40780-021-00213-y)
Supplement: Supplementary file 3 — Additional file 3. Funnel plots of the meta-analyses of fracture risk associated with anticholinergic burden: a) ARS, b) ACB, c) ADS, d) DBI-Ach. [file 40780_2021_213_MOESM3_ESM.docx]

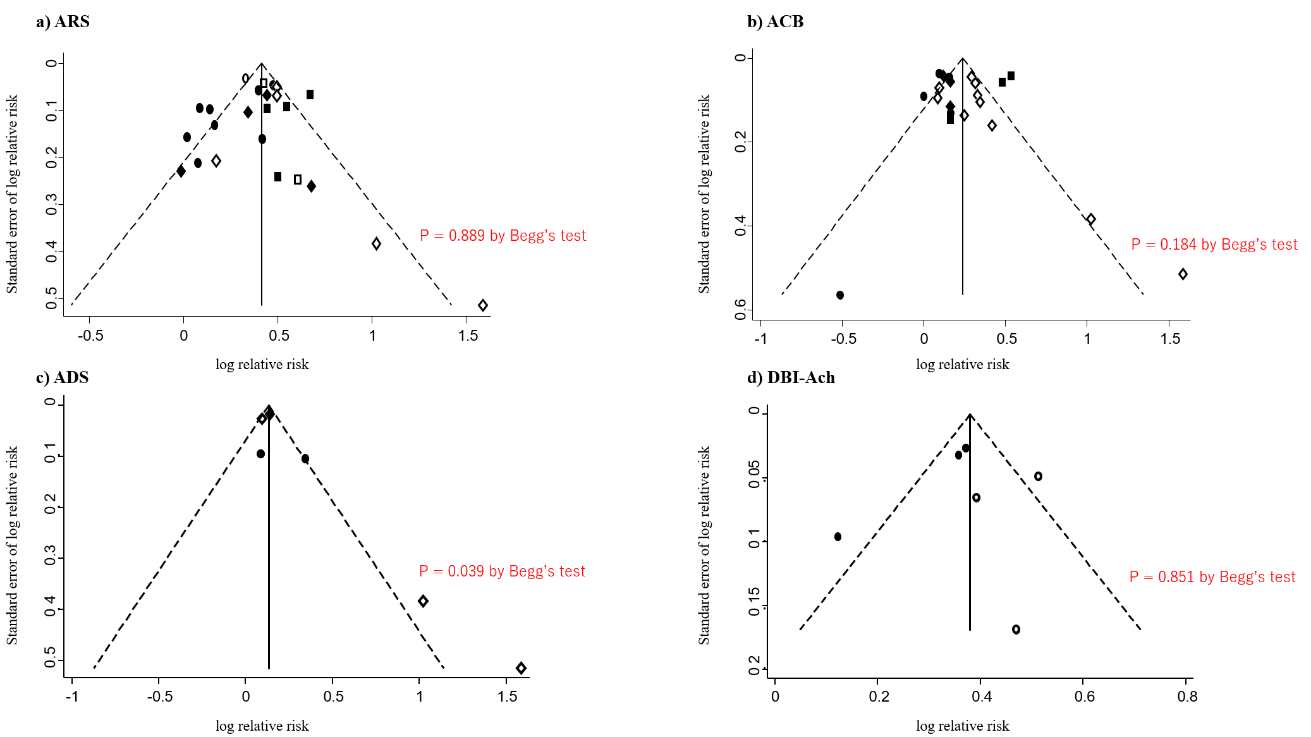


**Additional file 3** Funnel plots of the meta-analyses of fracture risk associated with anticholinergic burden: a) ARS, b) ACB, c) ADS, d) DBI-Ach.

ARS: anticholinergic risk scale, ACB: anticholinergic cognitive burden, ADS: anticholinergic drug scales, DBI-Ach: drug burden index-anticholinergic component

●ARS/ACB/ADS = 1 point or 0 < DBI-ach ≤ 0.5, ♦ARS/ACB/ADS = 2 points, ○ARS/ACB/ ADS = 1-2 point(s) or 0.5 < DBI-ach ≤ 1, ◊ ARS/ACB/ADS = 3 points, □ARS/ACB/ADS ≥ 3 points, ■ ARS/ACB/ADS ≥ 4 points
