# Supplementary material for: A meta-analysis of observational studies on anticholinergic burden and fracture risk: evaluation of conventional burden scales
Source: J Pharm Health Care Sci. 2021 Sep 1;7:30. doi: 10.1186/s40780-021-00213-y (PMC8408921; doi:10.1186/s40780-021-00213-y)
Supplement: Supplementary file 4 — Additional file 4. Thirty-five drugs included in both ARS and ACB. [file 40780_2021_213_MOESM4_ESM.docx]

| Drug name | Prescription drug in Japan | OTC drug  in Japan | Concordance between ARS and ACB scores | ARS  (points) | ACB  (points) | Concordance in fracture risk between ARS and ACB | Fracture risk category using ARS | Fracture risk category using ACB |
| --- | --- | --- | --- | --- | --- | --- | --- | --- |
| Amantadine | ✓ | - | ✓ | 2 | 2 | - | medium | low |
| Amitriptyline | ✓ | - | ✓ | 3 | 3 | - | medium/high | medium/low |
| Atropine | ✓ | - | ✓ | 3 | 3 | - | medium/high | medium/low |
| Benztropine* | - | - | ✓ | 3 | 3 | - | medium/high | medium/low |
| Chlorpheniramine | ✓ | ✓ | ✓ | 3 | 3 | - | medium/high | medium/low |
| Chlorpromazine | ✓ | - | ✓ | 3 | 3 | - | medium/high | medium/low |
| Cyclobenzaprine* | - | - | ✓ | 2 | 2 | - | medium | low |
| Dicyclomine | ✓ | ✓ | ✓ | 3 | 3 | - | medium/high | medium/low |
| Diphenhydramine | ✓ | ✓ | ✓ | 3 | 3 | - | medium/high | medium/low |
| Haloperidol | ✓ | - | ✓ | 1 | 1 | - | medium/low | low |
| Hydroxyzine | ✓ | - | ✓ | 3 | 3 | - | medium/high | medium/low |
| Hyoscyamine* | - | - | ✓ | 3 | 3 | - | medium/high | medium/low |
| Imipramine | ✓ | - | ✓ | 3 | 3 | - | medium/high | medium/low |
| Meclizine | - | ✓ | ✓ | 3 | 3 | - | medium/high | medium/low |
| Oxybutynin | ✓ | - | ✓ | 3 | 3 | - | medium/high | medium/low |
| Perphenazine | ✓ | - | ✓ | 3 | 3 | - | medium/high | medium/low |
| Promethazine | ✓ | ✓ | ✓ | 3 | 3 | - | medium/high | medium/low |
| Ranitidine | ✓ | - | ✓ | 1 | 1 | - | medium/low | low |
| Risperidone | ✓ | - | ✓ | 1 | 1 | - | medium/low | low |
| Thioridazine* | - | - | ✓ | 3 | 3 | - | medium/high | medium/low |
| Trazodone | ✓ | - | ✓ | 1 | 1 | - | medium/low | low |
| Trifluoperazine | ✓ | - | ✓ | 3 | 3 | - | medium/high | medium/low |
| Cetirizine | ✓ | ✓ | - | 2 | 1 | - | medium | low |
| Cimetidine | ✓ | - | - | 2 | 1 | - | medium | low |
| Clozapine | ✓ | - | - | 2 | 3 | - | medium | medium/low |
| Cyproheptadine | ✓ | - | - | 3 | 2 | - | medium/high | low |
| Desipramine* | - | - | - | 2 | 3 | - | medium | medium/low |
| Loperamide | ✓ | ✓ | - | 2 | 1 | - | medium | low |
| Loratadine | ✓ | ✓ | - | 2 | 1 | - | medium | low |
| Methocarbamol | ✓ | ✓ | - | 1 | 3 | ✓ | medium/low | medium/low |
| Nortriptyline | ✓ | - | - | 2 | 3 | - | medium | medium/low |
| Olanzapine | ✓ | - | - | 2 | 3 | - | medium | medium/low |
| Paroxetine | ✓ | - | - | 1 | 3 | ✓ | medium/low | medium/low |
| Quetiapine | ✓ | - | - | 1 | 3 | ✓ | medium/low | medium/low |
| Tolterodine | ✓ | - | - | 2 | 3 | - | medium | medium/low |

**Additional file 4** Thirty-five drugs included in both ARS and ACB.

AC: Anticholinergic, ACB: anticholinergic cognitive burden, ARS: anticholinergic risk scale, OTC: over the counter. *Not approved in Japan.
